# The Role of Selected Dietary Factors in the Development and Course of Endometriosis

**DOI:** 10.3390/nu15122773

**Published:** 2023-06-16

**Authors:** Anna Markowska, Michał Antoszczak, Janina Markowska, Adam Huczyński

**Affiliations:** 1 Department of Perinatology and Women’s Health, Poznań University of Medical Sciences, 60-535 Poznań, Poland; annamarkowska@vp.pl; 2 Department of Medical Chemistry, Faculty of Chemistry, Adam Mickiewicz University, 61-614 Poznań, Poland; michant@amu.edu.pl; 3 Gynecological Oncology Center, Poznańska 58A, 60-850 Poznań, Poland; jmarkmed@poczta.onet.pl

**Keywords:** endometriosis, polyphenols, vitamins, micronutrients, antioxidants

## Abstract

Endometriosis is a chronic disease with a complex, heterogeneous pathogenesis that affects about 10% of women of reproductive age, causing pain and leading to infertility. Treatment consists of administering pharmacological agents (resulting in a reduction of estrogen levels and inflammation), as well as the surgical removal of endometriotic lesions. Unfortunately, despite a wide range of available therapies, there is still a high recurrence rate after surgery. Consequently, it is necessary to improve the outcome of patients with endometriosis. In this context, there is growing interest in possible dietary modification to support or complement classic treatment options and even serve as a potential alternative to hormone therapy. In addition, a growing number of studies indicate positive effects of selected dietary factors on the development and course of endometriosis. This review article focuses on the potentially beneficial effects of compounds from the polyphenol group (curcumin, epigallocatechin gallate, quercetin, resveratrol), vitamins, and selected micronutrients on endometriosis. The results indicate the potential of the selected ingredients in fighting the disease. However, most of the studies have been performed on experimental animal models, with a smaller proportion looking at the actual effects of use among women. Therefore, well-designed studies are needed to assess the importance of a well-chosen diet and the effects of specific dietary factors on the health of women suffering from endometriosis.

## 1. Introduction

Endometriosis is a chronic hormone-dependent disease affecting ~10% of women of reproductive age, translating to nearly 200 million women worldwide [1,2,3,4]. This makes it one of the most common gynecological conditions of the reproductive period. As agreed by the International Working Group of AAGL, ESGE, ESHIRE, and WES in 2021, endometriosis is defined as a disease characterized by the presence of endometrial-like epithelium and/or stroma outside the endometrium and myometrium, often accompanied by inflammation [5]. The most common symptoms of this disease are severe pain during menstruation, intercourse (dyspareunia), or urination, but also bowel and bladder dysfunction [1,4,6]. In addition, chronic pelvic pain, which does not resolve with conventional treatment options, develops in ~30% of patients with endometriosis [4,7], while 30–50% of women are diagnosed with infertility [1,8,9], and nearly half suffer from anxiety or depression [1,10].

The pathogenesis of endometriosis is heterogeneous and involves the most diverse hormonal, genetic, immunological, as well as environmental factors. Among other things, its relationship to steroid metabolism, primarily estrogen, and the key role of 17β-estradiol in this process has been indicated [11]. Estrogens stimulate the epithelial-mesenchymal transition (EMT) in a variety of diseases, including ovarian cancer and breast cancer [12,13,14], leading to cells acquiring a mesenchymal phenotype. It is likely that they can also drive EMT in endometriosis, but it has been suggested that this is not crucial for the first stages of pathogenesis [15]. Estrogens decrease E-cadherin expression, which in turn increases the migratory and invasive potential of cells [16], and is characteristic of endometriosis. The phenomenon of progesterone resistance also appears to play an important role in the development of the disease. EMT may contribute to the downregulation of progesterone receptors in lesions, making them less sensitive to hormone therapy [17]. On the other hand, endometriosis may have a genetic origin. It has been established that gene mutations occur in about half the number of women suffering from this disease [18]. The most often met mutations concern KRAS, PIK3CA, and TTN [19]. Moreover, the potential involvement of different signaling pathways related to proliferation, apoptosis, migration, or angiogenesis, including MAPK/MEK/ERK, PI3K/AKT, mTOR, Rho/ROCK, or NF-κB, has been indicated [20]. Upregulation of several stemness-associated molecular markers [21,22,23] may suggest the participation of endometrium stem cells in the pathogenesis of endometriosis [24]. Moreover, the effects of immunological factors, pro-inflammatory and pro-angiogenic cytokines, e.g., TNFα, IL-1β, IL-6/8, or VEGF [2,6,25,26], may be significant.

Ultrasound and MRI (magnetic resonance imaging) are mainly used in the diagnosis of endometriosis, which aids in preoperative evaluation. Determination of CA-125 serum or numerous microRNAs is also helpful [27,28,29]. Treatment depends on the quality and size of the lesions and individual conditions. The most common treatment is hormonal therapy with danazol, gonadoliberin analogs, progesterone preparations, or contraceptives [6,20,30]. The aforementioned pharmacological treatments have similar therapeutic efficacy. The most recent drug approved by the FDA is elagolix, a GnRH antagonist [31]. The gold standard in the treatment of endometriosis is laparoscopy which allows both accurate diagnosis and eradication of the lesions [28], as surgery is the first-line therapy in symptomatic patients. Unfortunately, recurrences are common after both drug and surgical treatment; the overall recurrence rate was 21.5% and 40–50%, two and five years following surgery, respectively [32]. More than 45% of treated patients were not satisfied with the treatment method used [33]. Based on six randomized trials involving 675 patients and 16 cohort studies involving 3089 women after surgical treatment of endometriosis and adjuvant hormonal therapy, the actual effectiveness of hormonal treatment in preventing endometriosis recurrence among women after conservative surgery appears inconclusive [34].

Although the pathogenesis of endometriosis is still not fully explained, the disease may be related to oxidative stress and an abnormal increase in levels of reactive oxygen species (ROS) [35]. ROS activates programmed cell death mechanisms through interactions with various biological molecules [36]. Recent studies have highlighted the inverse (negative) correlation between the levels of ROS and antioxidants that causes a general inflammatory response in the peritoneal cavity, thus suggesting a pivotal role of oxidative stress in endometriotic pain and the development/progression of endometriosis [37,38,39]. Physiological factors can effectively neutralize the adverse effects of ROS; however, women with endometriosis may have an imbalance of both pro- and antioxidant molecules [35]. Moreover, the levels of antioxidants and thiols were significantly lower (*p* < 0.001) in the group of patients with endometriosis [40,41]. These reports indicate that low antioxidant levels may be integral to the pathogenesis of the disease and suggest that dietary intake of antioxidants (and other nutrients) should produce beneficial effects by reducing symptoms directly related to endometriosis.

The health-promoting properties of green tea, red wine, garlic, and fresh fruit have long been known, and their efficacy in disease prevention has been confirmed in numerous studies [42,43]. Medicinal plants may be a source of new drug candidates against endometriosis [44], and fruits or vegetables, rich in valuable nutrients, may have an impact on slowing the progression of the disease. There is growing interest among women in changing their diets to support or complement classical treatments for the disease and even as an alternative to hormone therapy [45,46,47,48,49,50].

In this review article, we focused on the potentially beneficial role of selected dietary factors from the group of polyphenols, vitamins, and micronutrients in inhibiting the progression of endometriosis. For this purpose, we ran a thorough search of the PubMed database for articles describing the role of the mentioned dietary components on the risk and development of the disease. A combination of words used as keywords was: “endometriosis” and “curcumin” or “epigallocatechin gallate” or “quercetin” or “resveratrol” for the polyphenol group; “endometriosis” and “vitamin C” or “vitamin D” or “vitamin E” for vitamins; and “endometriosis” and “calcium” or “zinc” or “nickel” for micronutrients. Articles that included the search keywords in the title and/or abstract were taken into examination. The lists of references of the examined publications were also checked in detail to identify eligible studies that could have been missed during the database search.

## 2. Polyphenols

Due to the unsatisfactory efficacy of available treatments and the chronic nature of endometriosis, more and more women are choosing to seek alternative treatments to control the progression of the disease or reduce the associated discomfort. Several studies have assessed the association between the administration of polyphenols, such as flavonoids or phytoestrogens, and the cancer risk in women [51,52]. Numerous similarities in cellular processes occurring between cancer and endometriosis have been noted, such as increased proliferation, invasion, and neoangiogenesis [53]. Given the very broad spectrum of health-promoting activities and low toxicity of polyphenols, including their anticancer potential, research into the protective effects of this group of compounds in terms of its potential against endometriosis, seems to be fully justified [44,48].

**Figure 1:**
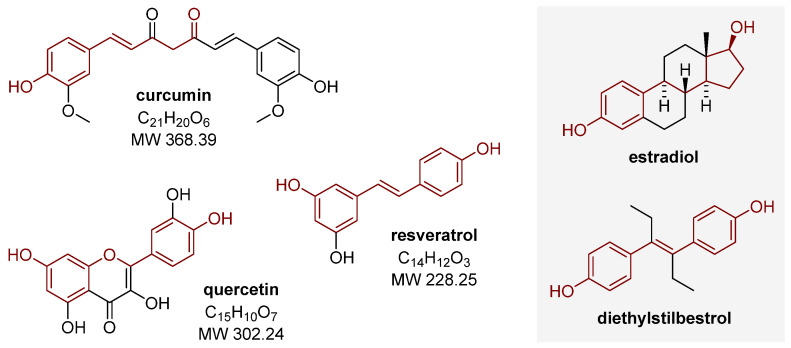
Structure of selected polyphenols and their similarity to estradiol and diethylstilbestrol.

**Figure 2:**
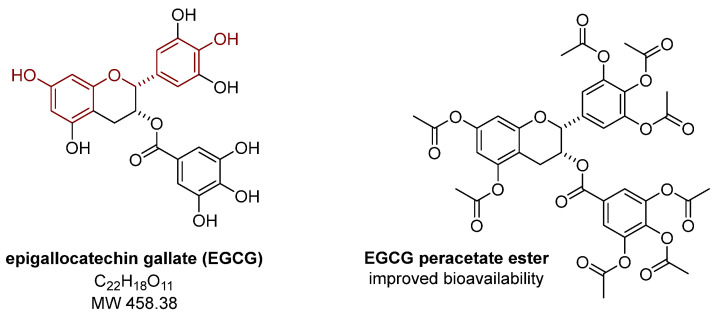
Structure of epigallocatechin gallate and its peracetate ester.

Polyphenols are an extensive group of biologically active compounds synthesized by plants. They are an integral part of the diet and are widely known for their properties, including antioxidant and anti-inflammatory properties that may be relevant to endometriosis. Curcumin, epigallocatechin gallate, quercetin, and resveratrol (Figure 1 and Figure 2) are among the most widely described compounds in the polyphenol group in terms of their possible positive effects on the risk and course of the disease. In addition to their antioxidant effects, the ability of polyphenols to affect estrogen receptors also appears to be important in the fight against endometriosis, which may be due to the structural similarity between these compounds and estradiol or the synthetic estrogen diethylstilbestrol (Figure 1) [48]. Studies have confirmed the role of resveratrol as an estrogen receptor agonist [47]. In addition, other polyphenols can also act through estrogen receptors to induce specific actions [47,48].

Signorile et al. [50] have presented the results of a study among endometriosis patients treated with a combination of natural ingredients administered in the form of supplements, compared to a control group receiving only a placebo. The use of a composition containing, among other things, curcumin and quercetin in the 30-person groups studied was associated with a significant reduction of adverse symptoms associated with endometriosis [50]. This included headaches, pelvic pain, and abnormal menstrual bleeding [50]. In addition, serum levels of prostaglandin E2 (PGE2) and CA-125 were found to be reduced in patients undergoing the treatment mentioned above [50]. On the other hand, Meresman et al. [47] have summarized the effects of the complex action of various plant-derived factors and natural treatment strategies in endometriosis, including curcumin, resveratrol, apigenin, puerarin, and cannabinoids. Their mechanism of action was pleiotropic and included effects on estrogen receptors, cyclooxygenase-2 (COX-2), interleukin (IL)-1/6, tumor necrosis factor α (TNFα), nuclear factor (NF)-κB, vascular endothelial growth factor (VEGF), and matrix metalloproteinases (MMPs), as well as apoptosis-related proteins [47]. Furthermore, a natural compound therapy alleviated chronic pelvic pain and reduced the recurrence rate and the extent of lesions [47]. According to the assessment with PRISMA (Preferred Reporting Items for Systematic Reviews and Meta-analyses), polyphenols, including curcumin, quercetin, and resveratrol, were found to have the potential for use in the treatment of endometriosis [54].

### 2.1. Curcumin

Turmeric (*Curcuma longa*) is a plant in the ginger family. It is native to Southeast Asia, mainly India, but it is grown commercially in many parts of the world. It is mainly used as a culinary spice but also for medicinal purposes. Curcumin (Figure 1) is one of the bioactive polyphenols contained in *Curcuma longa* [55]. Based on FDA evaluation, curcumin and its analogs have been approved as safe for oral use; an oral dose of 12 g per day is well tolerated. Studies suggest a potential role of curcumin as an anti-inflammatory, anti-tumor, or anti-aging agent [55]. A growing body of literature additionally points to a possible pharmacological effect of this compound in treating endometriosis, including a study by Singh et al. [56], which showed that curcumin could restore hemostasis in endometrial dysfunction. Vallée and Lecarpentier [57] have summarized the mechanisms of action of curcumin related to its effects on endometriosis: (i) anti-inflammatory activity—decreasing the activity of the NF-κB pathway at different stages of the disease, resulting in a decrease in the expression of inflammatory factors such as COX-2, TNFα, IL-1/6/8; (ii) decreasing the activity of VEGF, resulting in a decrease in PI3K/AKT pathway signaling associated with angiogenesis, proliferation, as well as apoptosis; (iii) antioxidant activity by decreasing MAPK/ERK pathway signaling related to oxidative stress; (iv) induction of programmed cell death by decreasing the activity of anti-apoptotic Bcl-2 proteins; and (v) reduction of MMPs expression, mainly MMP-9, leading to reduced development of endometriosis implants.

In an in vitro study conducted on human endometriotic stromal cells, it was noted that curcumin at a concentration of 50 µM increased the percentage of cells in the G1 phase and decreased those in the S phase [58]. The use of curcumin further decreased VEGF expression [58]. Much more numerous are reports of in vivo studies on the potential use of curcumin in endometriosis. In animal models, curcumin has been shown to affect endometriosis through the HIF signaling pathway, whose key targets are HIF-1α, IL-6, and VEGFA, resulting in improved local hypoxia and reduced inflammation [59]. The number of lesions, as well as the volume and degree of adhesions, were significantly smaller in the curcumin group compared to the control groups (*p* < 0.05) [59]. Another study showed up-regulation of MMP-2 activity by tissue inhibitor of metalloproteinases 2 (TIMP-2) in early endometriosis and an inhibitory effect of curcumin on endometriosis [60]. Swarnakar and Paul [61] described the ability of curcumin to inhibit both MMP-9 activity and the expression of this enzyme. In addition, attenuated MMP-9 activity was associated with reduced TNFα expression [61], indicating the anti-inflammatory properties of this polyphenol. Curcumin’s mechanism of action may also be based on the reduction of estradiol production [62]. According to the study’s authors, curcumin’s activation of estradiol genes may most likely be due to estrogen-like effects mediated by estrogen receptor α [62].

The use of curcumin alone (100 mg kg^−1^) and combined with the iron chelator deferoxamine led to reduced graft size and cell proliferation in an animal model [63]. Interestingly, despite curcumin’s ability to prevent the development of endometriosis, no significant changes were observed in specific factors in serum taken from rats with induced disease, including CA-125, homocysteine, and resistin levels [64]. The potential to inhibit angiogenesis, as expressed by its ability to decrease VEGF protein expression, has been demonstrated for curcumin in the previously mentioned in vitro studies [58] and in those conducted in an animal model [65].

On the other hand, various efforts have been made to improve the bioavailability, stability, specificity, and pharmacological properties of curcumin, also in the context of their possible use in the treatment of endometriosis, which includes, for example, obtaining transition metallo–curcumin complexes [66] and curcumin-loaded nanofibers [67].

### 2.2. Epigallocatechin Gallate

Epigallocatechin gallate (EGCG, Figure 2) is the main flavonoid in white, black, and especially green tea [68]. Due to its antioxidant, antiproliferative, and antiangiogenic properties, this polyphenol has caught the attention of scientists and physicians in the context of the possible prevention of various types of cancer, e.g., through the initiation of apoptosis and cell cycle arrest [69,70,71]. In addition, the properties of EGCG leading to a reduction of the size of endometriomas and selective inhibition of neovascularization in these lesions are also noteworthy [35].

Matsuzaki and Darcha [72], in a study conducted on samples from 55 women (45 endometriosis patients and 10 healthy females), have proved that EGCG could reduce the proliferation, migration, and invasion of endometriotic cells. In addition, using animal experiments—40 mice that received a single injection of proliferative endometrial fragments from 10 patient samples—EGCG was shown to prevent the progression of fibrosis in endometriosis [72]. Additionally, in an in vivo study, Ricci et al. [73] determined the effect of EGCG (and resveratrol) on the development of endometriosis. The study used 56 mice that underwent surgical induction of endometriosis, and then, 15 days after surgical intervention, therapy was initiated using polyphenols, which was continued for another four weeks [73]. In both cases, reduced mean numbers and volumes of established lesions were observed (*p* < 0.005) [73]. Although both used treatments proved effective in terms of reducing cell proliferation, vascular density, or an increase in the proportion of apoptosis, the results obtained for EGCG (*p* < 0.05) were less significant than those obtained for resveratrol (*p* < 0.01) [73].

On the mechanistic side, EGCG can affect the expression of E-cadherin at the cell membrane and reduce the DNA methylation status of the E-cadherin promoter region, resulting in the inhibition of endometriotic lesion growth [74]. Furthermore, EGCG can also inhibit blood vessel formation by inhibiting VEGFC/VEGFR2 expression and signaling pathway, as shown in both in vitro and in vivo studies [75]. On the other hand, to increase the bioavailability of EGCG, this polyphenol was converted into a peracetate ester (Figure 2) and then tested in vivo [76]. By doing so, it has been demonstrated that chemical modification of EGCG can improve the bioavailability and the antioxidant and anti-angiogenic properties of the obtained derivatives compared to the native structure [76].

### 2.3. Quercetin

Quercetin (Figure 1) is a naturally occurring hydroxyl derivative of flavone. This flavonol is present in various fruit and vegetables, including apples, berries, onions, cauliflower, and chili peppers. Quercetin exhibits activity against cancer cells through various mechanisms [47,77]. Based on the evaluation of the PRISMA study, quercetin was also found to show activity against endometriosis according to the exact mechanisms of curcumin [54].

Quercetin inhibited the proliferation of endometriosis-regulating cyclin D1 and its target microRNAs in in vitro and in vivo studies [77]. Mechanistically, this polyphenol induced cell apoptosis with DNA fragmentation, loss of mitochondrial membrane potential, and ROS generation; the effects were accompanied by the down-regulation of extracellular signal-regulated kinase 1/2 (ERK1/2), P38 MAPK (mitogen-activated protein kinase) and AKT signaling molecules [77]. It was shown in animal models that a combination of quercetin and metformin—a drug belonging to the biguanide group, commonly used as an oral antidiabetic drug for the treatment of type 2 diabetes—had a positive effect on the size of endometrial implants, as well as the expression levels of mammalian target of rapamycin (mTOR) and autophagy marker genes in the ectopic endometrium [78]. Compared to a group of rats receiving a placebo, quercetin (100 mg kg^−1^), the hormone drug danazol (36 mg kg^−1^), and a combination of both compounds significantly reduced implant size, with no apparent changes observed between control groups [79]. Another study using rats concluded that the mechanism of quercetin’s inhibition of ectopic endometrial growth might involve a decrease in serum levels of follicle-stimulating hormone (FSH) and luteinizing hormone (LH), followed by a decrease in local estrogen, resulting in the disappearance of the ectopic endometrium [80].

In a study of 33 women with a clinical diagnosis of endometriosis, Fadin et al. [81] observed a marked reduction of pain (*p* < 0.001) in patients, as well as the use of much lower amounts of non-steroidal anti-inflammatory drugs resulting from supplementation with 200 mg of quercetin, 210 mg of dry turmeric extract, and 150 mg of acetylcysteine over two months. In doing so, no significant side effects were reported [81].

### 2.4. Resveratrol

Resveratrol (Figure 1) is a natural polyphenol, a hydroxyl derivative of stilbene, present in many plant species, including legumes, berries, and peanuts, but the richest sources of this compound are grapes and red wine. Resveratrol exhibits a number of important health-promoting properties, including cardioprotective, anti-inflammatory, antioxidant, antiangiogenic, antimicrobial, and anticancer properties [82]. In addition, it has been suggested that resveratrol therapy, in particular in the context of its anti-inflammatory effects [83], may contribute to the prevention of endometriosis, as well as the alleviation of symptoms of the disease. It may also inhibit the development of new microvessels in endometriotic lesions by reducing endothelial cell proliferation [84].

Several in vitro studies to determine the effects of resveratrol use on endometriotic cells have shown proapoptotic effects, as well as limitation of the proliferation and invasive nature of these cells [85,86,87,88,89], which has been linked to, among other things, the polyphenol’s inhibitory effect on insulin-like growth factor 1 (IGF-1) and hepatocyte growth factor (HGF) expression [87]. In vitro studies of stromal cells and endometrial epithelium have shown that resveratrol impairs molecular mechanisms—reduces cell viability and migration, decreases MMP-2 activity, and affects stem cells, as manifested by changes in the expression of their markers (NANOG, OCT-4, SOX-2) [90]. Resveratrol (10–30 µM) caused a concentration-dependent reduction of human endometrial stromal invasiveness by nearly 80% (*p* < 0.0001) [91]. In the case of human ectopic endometrial lining cells, a reduction of proliferation capacity and invasion potential was noted in response to treatment with resveratrol, and the number of cells in early apoptosis increased [92]. In turn, the observed activation of peroxisome proliferator-activated receptor α (PPARα) may be a potential contributor to recovery from endometriosis [92]. Treatment with high concentrations of resveratrol (100 µM) resulted in decreased expression of VEGF, transforming growth factor β (TGF-β), and MMP-9 in endometrial lining cells [93].

Studies conducted on in vivo models of endometriosis confirmed both the proapoptotic and anti-invasive role of resveratrol and its inhibitory effect on angiogenesis, as evidenced by a decrease in the expression of various pro-inflammatory, pro-invasive, and pro-angiogenic factors [94,95,96], including IL-6/8, VEGF, and monocyte chemoattractant protein 1 (MCP-1). Amaya et al. [95] examined the dose-dependent effects of resveratrol on the endometrium. In addition to exhibiting antioxidant properties, the compound acts as a phytoestrogen, producing different effects depending on the concentration; at low concentrations, it acted agonistically. In contrast, at high concentrations, it acted antagonistically [95]. Given that endometriosis is a disease that is related to estrogen levels, it has been shown that high concentrations of resveratrol can reduce the proliferation of human endometrial xenografts in mice [95]. Studies in a mouse model of endometriosis further demonstrated that metastasis associated 1 (MTA1) and zinc finger E-box binding homeobox 2 (ZEB2) were up-regulated in ectopic tissues, while polyphenols inhibited ectopic lesion growth and MTA1/ZEB2 expression [97].

The use of resveratrol on rats also resulted in a positive therapeutic effect [98,99]; a transcriptional analysis showed that using polyphenol on model rats with endometriosis resulted in changes in PPAR, MAPK, and PI3K/AKT signaling pathways [98]. Bayoglu Tekin et al. [100] tested the effects of resveratrol use on an animal model in experimentally induced endometriosis, with the effects of resveratrol compared to those exhibited by leuprolide acetate, a synthetic gonadoliberin analog, used to treat endometriosis. A reduction of implant size, a decrease in MMP-2/9 expression, and lower levels of IL-6/8 and TNFα induced by resveratrol (30 mg kg^−1^ administered intramuscularly over two weeks) led the study’s authors to conclude that the polyphenol administered alone could be a potential agent for treating the disease [100]. In addition, the efficacy of resveratrol in combination with statins, such as atorvastatin [101] and simvastatin [102], has also been proven.

Maia et al. [103] evaluated the efficacy of resveratrol in treating pelvic pain and painful menstruation among 12 women with endometriosis. Adding 30 mg of resveratrol for two months to oral hormonal contraception resulted in complete resolution of pain in more than 80% of the patients, which was associated with inhibiting two enzymes—aromatase and COX-2 [103]. The study’s authors suggest that the addition of resveratrol to contraceptives for women with endometriosis may enhance the therapeutic effects of these drugs [103]. However, a subsequent randomized clinical control study of 44 women did not confirm the results described above [104]. In women with laparoscopically confirmed endometriosis, the addition of 40 mg of resveratrol in two cycles of hormonal contraception (levonorgestrel plus ethinylestradiol) was no more effective than placebo [104].

In a randomized study, resveratrol was shown to modify the inflammatory process in women with endometriosis at the MMP level [105]. In a group of 34 patients, half of whom received resveratrol at a dose of 400 mg for 12–14 weeks, and half received a placebo, there was a reduction of MMP-2/9 levels in the polyphenol-treated group and after surgical intervention [105]. The same group of researchers evaluated in a later study the effect of resveratrol on angiogenesis, a vital process accompanying the development of endometriosis [106]. In addition, women with an advanced form of the disease (stage III and stage IV) experienced a reduction of VEGF and TNFα expression after using resveratrol (400 mg) for 12–14 weeks compared to a placebo-treated control group [106].

## 3. Vitamins

The potential beneficial effect of supplementation with antioxidants on the healing process of endometriosis has been described by many researchers; however, clear and fully convincing evidence in this regard is still needed [107]. Additionally, data on the possible counteracting effects of oxidative stress by vitamin C and vitamin E among women with endometriosis, remain inconsistent [108,109]. Similarly, it is uncertain whether serum vitamin D levels are altered among endometriosis patients [110]. Nevertheless, given the possible beneficial effects of vitamins in preventing and treating selected female malignancies, including breast, cervical, endometrial, and ovarian cancers [111,112,113,114], research into the contribution of selected vitamins to endometriosis seems warranted.

### 3.1. Vitamin C and Vitamin E

Vitamin C plays a significant role in fighting free radicals due to its antioxidant properties. Vitamin C is an exogenous vitamin, so it is necessary to supply it with food or through appropriate supplementation. Fruit and vegetables, especially rosehips, black currants, strawberries, citrus, parsley, or peppers, are excellent sources of vitamin C. Vitamin E, in turn, is the collective name for a group of fat-soluble compounds with antioxidant activity. Nuts, seeds, and vegetable oils are among the primary sources of this vitamin; significant amounts are also available in green leafy vegetables and cereals. A 2009 study found that women with endometriosis had 30% and 40% lower intakes of vitamin C and E, respectively, compared to those who did not have the disease [108]. However, after three months of an antioxidant-rich diet, higher peripheral concentrations of supplemented vitamins were found [108].

Hoorsan et al. [115], based on their findings in mice, concluded that vitamin C could improve ovarian function and reduce the induction and growth of endometrial grafts. Similar conclusions were reached in a study on rats. It was proven that intravenously administered vitamin C could inhibit the induction of endometriotic implants and the regression of their volume [116]. In addition, the effect of the use of vitamin C and vitamin E on the expression and production of the VEGF gene in the peritoneal macrophages of women who have endometriosis, compared to a control group, was determined [117]. It was found that both vitamins, at different incubation times and concentrations, could alter the expression of the VEGF gene but did not affect its production [117].

In contrast, a randomized, triple-blind, placebo-controlled study from 2021 found that vitamin C and vitamin E supplementation could reduce systemic oxidative stress markers among patients with endometriosis [118], which was consistent with the results of previous studies [119]. The severity of pelvic pain, painful menstruation, and dyspareunia decreased after eight weeks of taking these vitamins (*p* < 0.001) [118]. However, there was no significant improvement in the pregnancy rate, i.e., the percentage of pregnancies ending in a live and healthy delivery, during or after therapy; the pregnancy rates were 19% and 12% in the supplementation and placebo groups, respectively [119]. Positive effects of vitamin C and vitamin E supplementation on the reduction of endometriosis symptoms compared to the placebo-treated control group were further found by Santanam et al. [120]. A prospective cohort study that included 1383 cases of laparoscopically confirmed endometriosis found that the intake of vitamin C and vitamin E (as well as thiamine and folic acid) from food is admittedly inversely related to the risk of endometriosis, but, according to the authors of this study, the mechanisms of protection may not be directly related to the vitamins mentioned above, but rather due to the presence of other essential nutrients in the diet as well [121].

### 3.2. Vitamin D

Vitamin D plays a role in regulating blood calcium concentration and modulating the immune system. Oily fish, fish fats, and supplements are essential sources of this vitamin, but the best way to obtain it is through dermal synthesis occurring under ultraviolet B radiation. Since endometriosis is a chronic inflammatory disease that may result from immune system dysfunction, it seems an intriguing line of research to determine the effect of the resistance modulator, vitamin D, on endometriosis, especially in the context of the vitamin’s association with various autoimmune and inflammatory diseases.

A meta-analysis conducted in 2020 found that low levels of vitamin D may correlate with an increased risk of being diagnosed with endometriosis, as well as a possible increase in symptoms of the disease [110]. Similar correlations have been found in other studies [122,123,124,125,126]; for example, a correlation between serum vitamin D levels in women of childbearing age diagnosed with single ovarian endometriosis and lesion size [127]. There are, however, studies that do not support the above reports [128,129]. Further, data on the regression of endometriosis with vitamin D still need to be more conclusive because significant differences are observed in the results obtained in cell studies and animal models compared to the effects of vitamin D use by women [130,131]. The results of a case–control study by Buggio et al. [132] did not confirm an association between serum 25-hydroxyvitamin D levels and the different phenotypes of endometriosis.

In in vitro tests on cells taken from 25 women with endometriosis and 20 women without endometriosis, the use of 1,25-dihydroxy vitamin D resulted in, among other things, increased adhesion (*p* = 0.0013–0.042), decreased potential for invasion (*p* = 0.026–0.031), and proliferation (*p* = 0.0013–0.039) of eutopic and ectopic endometriosis stroma cells [133]. In addition, a decrease in IL-6 production was noted, compared to no significant effect on IL-8 production [133]. In contrast, a decrease in IL-8 expression under the influence of 1,25-dihydroxy vitamin D was noted by Miyashita et al. [126]. By modulating IL-17 expression in endometriotic lesions, vitamin D at an optimal dose of 24 IU caused inhibition of lesion development in a mouse model of endometriosis [134]. Beneficial effects of vitamin D in rat studies were, in turn, noted by Abbas et al. [135]; vitamin D treatment induced fibrosis as well as apoptosis in the stroma. In another animal study, however, vitamin D was less effective against endometriosis than omega-3 polyunsaturated fatty acids; when vitamin D was used, only a reduction of IL-6 levels was observed [136], which was the same as the findings reported earlier [133].

A randomized, controlled trial on a group of 60 women with endometriosis showed that administering vitamin D at a dose of 50,000 IU every two weeks for 12 weeks could cause a reduction of perceived pelvic pain [137]. Using the same dose of vitamin D weekly for 12–14 weeks in women with an advanced form of endometriosis (stage III/IV) resulted in a change in β-catenin protein activity in endometriotic cells [138]. Similar observations of reduced pain were observed among young women (12–25 years old) but were similar to those in the control group [139]. On the contrary, after ablative surgery for endometriosis, vitamin D had no significant effect on reducing pelvic pain and/or painful menstruation [140].

## 4. Micronutrients

A well-balanced diet containing the necessary amount of micronutrients is crucial to the proper functioning of the body. Individual micronutrients affect many biological processes, including inflammation and immunity, which, if abnormal, can contribute to the development of lesions. Some studies have shown that the level of micronutrients in the serum of patients with endometriosis differs from that in healthy women, suggesting that this factor may be involved in the heterogeneous pathogenesis of the disease. However, the amount of data in the literature on the role of micronutrients in the risk of endometriosis is severely limited compared to those described in earlier chapters on polyphenols or vitamins. Nevertheless, the effects of calcium, zinc, or nickel on the pathogenesis of the disease or the symptoms resulting from it have been analyzed by several researchers.

A systematic review of the literature in the area, as well as a meta-analysis of observational studies conducted using electronic databases in the PROSPERO study, showed that dietary factors playing a role in the etiology of endometriosis might be related to the calcium (and vitamin D) content of the foods consumed [141]. They may decrease the expression of IGF-1, TGF-β, TNFα, and IL-6 [141]. A possible role of zinc deficiency, a micronutrient with important functions in the body—regulation of cell growth, hormone release, and immune response—in the etiopathogenesis of endometriosis has also been described [142]. In a group of 42 patients with endometriosis, the average serum zinc concentration was 1010 ± 59.24 µg L^−1^, while in 44 healthy women, the level of this micronutrient was slightly higher (1294 ± 62.22 µg L^−1^) [142]. Lai et al. [143] have confirmed that adequate zinc levels may be associated with decreased risk of endometriosis (OR 0.39, 95% CI 0.18–0.88). In turn, a meta-analysis of electronic databases published in 2022 found that supplementation with selected dietary components (e.g., vitamins A, C, D, or E, omega-3/6 acids, curcumin, quercetin) and complete personal dietary modification (e.g., gluten-free), as well as the exclusion of specific dietary components, including nickel, had a positive effect on endometriosis-related symptoms, including pain perception, in the majority of patients studied [45].

## 5. Summary

Endometriosis is a complex gynecological disease of women of reproductive age that causes chronic pain, painful menstruation, and dyspareunia. The heterogeneity of the disease makes accurate diagnosis and treatment a clinical challenge. Current therapies, including hormone therapy and pain medications, are often ineffective, are associated with unwanted side effects that limit their long-term use, and may reduce fertility. Laparoscopy makes it possible to eradicate lesions, but numerous recurrences are observed after both drug and surgical treatment. Therefore, searching for new diagnostic and therapeutic options is crucial.

Diet and nutrition are fundamental to health. Numerous studies have provided solid evidence of the positive health effects of a diet rich in fruits and vegetables due to the presence of valuable nutrients. Importantly, dietary factors can significantly impact estrogen action or inflammatory processes, which are directly related to the pathophysiology of endometriosis. The oxidative stress status may represent the key to controlling the disease. In this context, the anti-inflammatory, antioxidant, or antitumor potential of selected polyphenols or vitamins can be used as an inexpensive and readily available means for preventing and treating endometriosis. The potential beneficial effects of these agents have been determined mainly based on results from animal studies and included, among other things, a reduction of the size and number of lesions. However, it should be considered that results obtained in a mouse or rat model of endometriosis may only partially correlate with those in humans. Nevertheless, several studies conducted with women have indicated the alleviation of pain and other symptoms of endometriosis, especially after using selected compounds from the polyphenol group.

The studies described in the scientific literature highlight the great therapeutic potential inherent in the dietary factors discussed in this review article. However, there is a need for in-depth, well-designed studies, primarily randomized clinical trials, to clarify the role of diet in endometriosis and to assess the actual prophylactic and/or therapeutic utility of its components. In many cases, conflicting results have been obtained, and discrepancies between different studies may be due to differences in study design, patient selection, and analytical methods. In addition, the possible side effects of using individual compounds should be considered, especially at high concentrations. In such cases, it will also be necessary to determine the minimum effective and maximum safe doses for women with endometriosis and exclude and/or treat potential risk factors. The relatively low bioavailability of selected compounds, for example, curcumin or EGCG, may also be a limitation, making their clinical use difficult.

Nevertheless, including an appropriate diet rich in polyphenols, vitamins, or micronutrients as the standard treatment protocol may contribute to greater control of endometriosis. We must emphasize that the intake of selected nutritional factors cannot replace standard therapy but should support or supplement it. Furthermore, in-depth clinical and control studies on evaluating individual supplements in combination with standard treatments are required.

## Data Availability

Not applicable.
